# Intravitreal pro-inflammatory cytokines in non-obese diabetic mice: Modelling signs of diabetic retinopathy

**DOI:** 10.1371/journal.pone.0202156

**Published:** 2018-08-22

**Authors:** Odunayo O. Mugisho, Ilva D. Rupenthal, David M. Squirrell, Sarah J. Bould, Helen V. Danesh-Meyer, Jie Zhang, Colin R. Green, Monica L. Acosta

**Affiliations:** 1 Buchanan Ocular Therapeutics Unit, Department of Ophthalmology and the New Zealand National Eye Centre, University of Auckland, Auckland, New Zealand; 2 Department of Ophthalmology and the New Zealand National Eye Centre, University of Auckland, Auckland, New Zealand; 3 School of Optometry and Vision Science and the New Zealand National Eye Centre, University of Auckland, Auckland, New Zealand; University of Florida, UNITED STATES

## Abstract

Diabetic retinopathy is a vascular disease of the retina characterised by hyperglycaemic and inflammatory processes. Most animal models of diabetic retinopathy are hyperglycaemia-only models that do not account for the significant role that inflammation plays in the development of the disease. In the present study, we present data on the establishment of a new animal model of diabetic retinopathy that incorporates both hyperglycaemia and inflammation. We hypothesized that inflammation may trigger and worsen the development of diabetic retinopathy in a hyperglycaemic environment. Pro-inflammatory cytokines, IL-1β and TNF-α, were therefore injected into the vitreous of non-obese diabetic (NOD) mice. CD1 mice were used as same genetic background controls. Fundus and optical coherence tomography images were obtained before (day 0) as well as on days 2 and 7 after intravitreal cytokine injection to assess vessel dilation and beading, retinal and vitreous hyper-reflective foci and retinal thickness. Astrogliosis and microgliosis were assessed using immunohistochemistry. Results showed that intravitreal cytokines induced vessel dilation, beading, severe vitreous hyper-reflective foci, retinal oedema, increased astrogliosis and microglia upregulation in diabetic NOD mice. Intravitreal injection of inflammatory cytokines into the eyes of diabetic mice therefore appears to provide a new model of diabetic retinopathy that could be used for the study of disease progression and treatment strategies.

## Introduction

Diabetic retinopathy (DR) is the most common microvascular complication of diabetes leading to vascular breakdown in the retina. In 2014, approximately 370 million people suffered from diabetes worldwide [1]. Remarkably, this number is expected to double by 2030 meaning that 1 in 5 adults will suffer from the disease. DR is primarily a vascular disease characterised by endothelial cell [2] and pericyte loss [3] as well as blood retinal barrier (BRB) breakdown [4,5]. The loss of vascular integrity can trigger retinal neovascularisation, the formation of new but leaky blood vessels (proliferative DR). Eventually, there is fluid build-up within the retina that can affect the macula leading to diabetic macular oedema (DME). While the vascular pathology and progression associated with DR is well understood, the underlying molecular mechanisms are still enigmatic.

A key limitation to research into DR intervention strategies is the lack of comprehensive models that mimic the various stages of the disease. One possible reason is that most animal models used are diabetes, i.e., hyperglycaemia-only, models that do not account for the role that inflammation may play in the development of diabetic retinopathy in the eye. It is currently believed that hyperglycaemia causes inflammation; therefore, most of the literature assumes that DR is a result of prolonged hyperglycaemia [6]. However, recent clinical studies have shown that there is a high correlation between the incidence of diabetes and inflammation of the uveal tract (called uveitis) suggesting that inflammation may be a DR trigger rather than a consequence of hyperglycaemia [7–9]. Studies have also shown that there is an increase in pro-inflammatory cytokine levels in the vitreous of DR patients compared to controls [10–12]. Furthermore, a recent retrospective study found that uveitis in diabetic patients progressed to more severe ocular complications and poorer vision [13]. These studies, taken together, support the need for in-depth investigation into the relationship between diabetes and inflammation in DR.

We therefore investigated whether a model of DR can be established by introducing pro-inflammatory cytokines into the vitreous of hyperglycaemic diabetic mice based upon the hypothesis that intravitreal injection of pro-inflammatory cytokines into the eyes of diabetic mice could induce DR. As a result, we investigated the effect of the inflammatory cytokines, IL-1β and TNF-α, in non-obese diabetic (NOD) mice. NOD mice spontaneously develop type I diabetes due to T-cell mediated autoimmune destruction of their β-islet cells [14]. While some older studies have shown loss of retinal microvasculature and decreased retinal perfusion in NOD mice [15,16], signs of proliferative DR have not been reported in this model. Therefore, using *in vivo* fundus and optical coherence tomography (OCT) imaging, we evaluated the added contribution of inflammation to the development of DR assessed by changes in the retina, vitreous and optic nerve.

## Materials and methods

### Animals

Female, 15-week old NOD mice (NOD/ShiLtJ; Stock No: 001976) obtained from Jackson Laboratory (Bar Harbor, ME, USA) and CD1 mice (Crl:CD1(ICR); Strain code: 022) obtained from Charles River Laboratories (Wilmington, MA, USA), were used in this study. A group of Swiss mice derived from a non-inbred stock in the laboratory of Dr. de Coulon, Centre Anticancereux Romand, Lausanne, Switzerland served as progenitors for both NOD and CD1 mouse strains [17]. CD1 mice were used as controls in this study as they have the genetic background for NOD mice. Female NOD mice were selected as they are more likely to develop hyperglycaemia compared to their male counterparts. Animals were bred and housed in the Vernon Jansen Unit at the University of Auckland under normal cyclic light conditions (12 h light: 12 h dark) and had access to food and water *ad libitum*. All procedures were performed in accordance with the local welfare legislations and approved by the University of Auckland Animal Ethics Committee (AE1787 approved on 16 Sept 2016). Experiments were conducted in mice anaesthetised by an intraperitoneal injection of ketamine (50 mg/kg) and domitor (0.5 mg/kg). Following *in vivo* ocular assessments, mice were awakened by an intraperitoneal injection of atipamezole (5 mg/kg). Baseline ocular assessment including ocular function was conducted prior to cytokine injections. Twelve eyes (6 mice) from each mouse strain were intravitreally injected with pro-inflammatory cytokines while six eyes (3 mice) from each mouse strain receiving intravitreal phosphate buffer saline (PBS) injections were used as controls.

### Length, weight and body mass index (BMI) determination

Mice were weighed before and 1, 2, and 7 days after intravitreal injection. Weight measurements were performed consistently at the same time of the day. The mouse length was measured from the tip of the snout to the base of the tail using a ruler. The body mass index (BMI) was calculated by dividing the mass of the mouse (g) by the square of the animal length (mm^2^). In comparing the blood glucose levels between CD1 and NOD mice, the NOD mouse glucose levels were not normally distributed as confirmed by a Shapiro-Wilk normality test. As a result, nonparametric Mann-Whitney test was used to analyse statistical differences in blood glucose between the two mouse strains.

### Non-fasting blood glucose determination

Non-fasting blood glucose levels were determined immediately after anaesthesia. A 25G needle (BD Bioscience, CA, USA) was used to prick the lateral tail vein. Blood glucose measurements were carried out using a glucose meter (Freestyle Optium H Glucometer, UK) and test strips (FreeStyle Optium, UK). Readings above the upper limit of the equipment (27.8 mmol/L) were treated as 27.8 mmol/L during data analysis. After collection of the blood sample, pressure was applied to the tail for a few seconds until bleeding stopped.

### Intravitreal injection of pro-inflammatory cytokines

Mouse recombinant IL-1β (#RMIL1BI, Thermo Fisher Scientific, MA, USA) and mouse recombinant TNF-α (#RMTNFAI, Thermo Fisher Scientific) were intravitreally injected into both eyes of CD1 (non-diabetic) and NOD (diabetic) mice. A pilot study with three different cytokine concentrations (50, 100 and 500 ng/mL per cytokine) was carried out prior to this study to determine the optimum cytokine dose required to elicit moderate ocular effects. Control animals received an intravitreal injection of PBS. A volume of 1 μL of cytokines at a concentration of 500 ng/mL each was injected into the vitreous using a 10 μL Hamilton syringe attached to a 30G ½″ needle (Terumo Medical Corporation, NJ, USA). Injections were performed in anaesthetized mice using a dissection microscope to visualise the needle and avoid damage to the lens. The injection was consistently performed on the temporal side immediately posterior to the limbus at the corneal-scleral intersection.

### Fundoscopy and optical coherence tomography (OCT) imaging

Mice were anaesthetised and pupils were dilated with 1% tropicamide (Minims, UK). The cornea was kept moist using a lubricating eye gel (GenTeal^®^ Gel; Alcon, Switzerland) and the Micron IV imaging system (Phoenix Research Labs; CA, USA) was used to obtain both fundus and image-guided OCT images before (day 0) as well as on days 2 and 7 after intravitreal cytokine injection. Vessel dilation and beading were assessed from fundus images. Vessel dilation was qualitatively defined as an increase in vessel diameter compared to day 0, before treatment. Vessel tortuosity was qualitatively assessed as a loss of vascular rigidity and increase in vessel ‘waviness’. Vessel beading was qualitatively defined as the presence of inconsistent vascular tone within a blood vessel compared to day 0. The researcher was masked to the strain and treatment received in order to reduce any bias. OCT images were also taken from the periphery of the eye cup, where lesions would mostly occur in the human condition [18]. Representative images of central and peripheral retina (five superiorly and three inferiorly to the optic nerve head (ONH)) spanning nasal and temporal regions were taken.

### Assessment of vitreous and retinal hyper-reflective foci (HRF) and severity grade

Hyper-reflective foci (HRF) observed in OCT scans of retina and vitreous in human patients represent migratory inflammatory infiltrating cells [19]. In a masked experiment, one of the researchers qualitatively assessed OCT images to evaluate the presence of HRF from day 0 through to day 7 in all mice. OCT images were obtained in a predetermined raster sequence with each OCT image being spaced 0.25 mm apart. The raster pattern used thus generated five OCT images located superior to the ONH, one located on the ONH and three inferior to the ONH. Each eye was scanned on three separate days (0, 2 and 7) giving a total of 27 OCT scans per group. The presence of HRF in the vitreous was assessed against a pre-determined severity scale. A severity grade for each eye was allocated if signs were seen in at least four out of the nine OCT images. The severity of vitreous HRF was measured as integrated vitreous HRF ranking which was determined by multiplying the vitreous HRF grade by the total number of eyes with vitreous HRF within the group. The presence of intra-retinal HRF in OCT scans were also noted but not graded.

### Calculation of retinal thickness from OCT images

**Fig 1:**
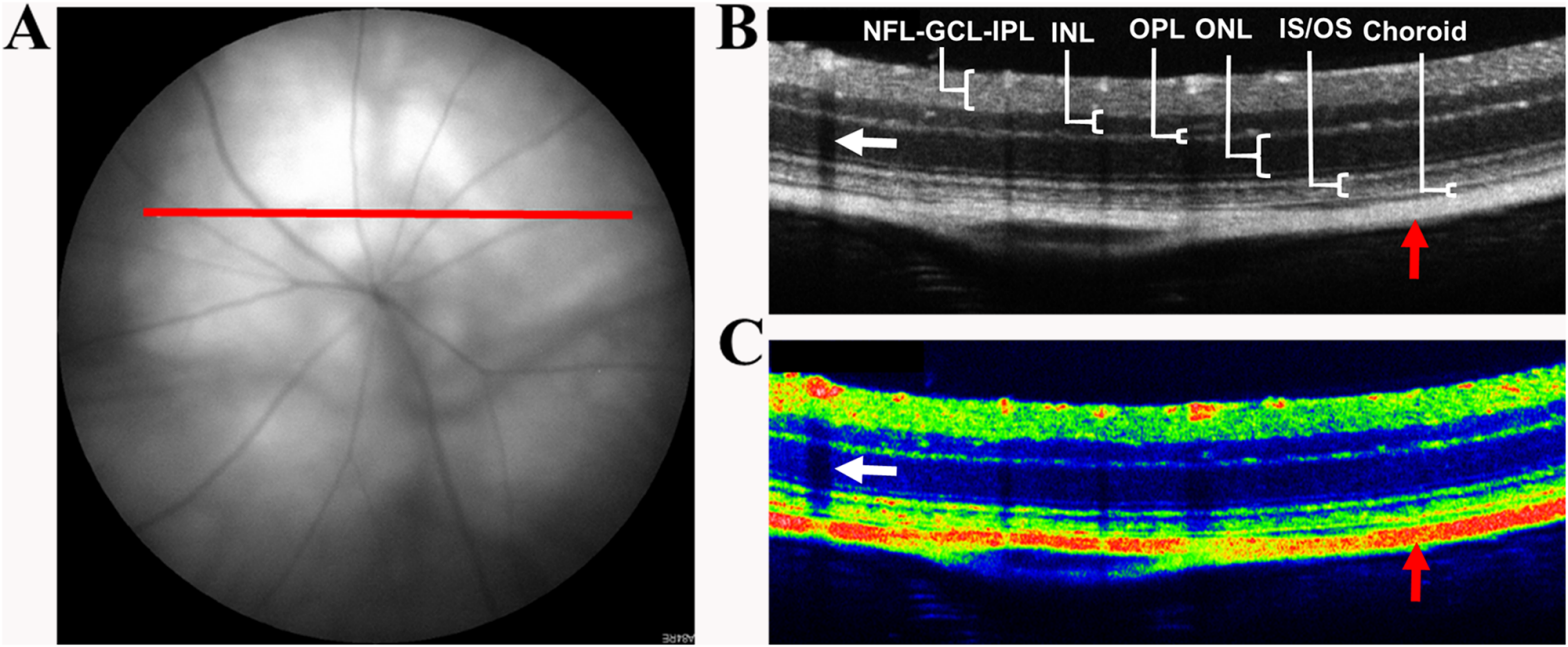
(A) Fundus image showing the position (red line) at which OCT scans were taken to quantify retinal layer thicknesses. (B) OCT image obtained from the position indicated by the red line in (A) showing the different retinal layers quantified. (C) Pseudo-colour OCT image showing the log of the backscattered light intensity. Fluid-filled areas and blood vessel shadows (white arrows) appear hypo-reflective and therefore black in colour OCT images while cell-dense and hyper-reflective areas range in colour from blue to red (red arrows). NFL = Nerve fibre layer; GCL = Ganglion cell layer; IPL = Inner plexiform layer; INL = Inner nuclear layer; OPL = Outer plexiform layer; ONL = Outer nuclear layer; IS/OS = Inner segment/outer segment.

The thickness of retinal layers was measured from OCT images between the nerve fibre layer (NFL) and the choroid using the ‘draw line’ and ‘measure’ functions in ImageJ software version 1.46r (National Institutes of Health, MD, USA). Due to the nature of OCT imaging in mice, it was difficult to differentiate between NFL, ganglion cell layer (GCL) and inner plexiform layer (IPL) and all three layers were thus quantified together. Layer thicknesses were acquired from OCT images taken consistently approximately 0.25 mm superiorly to the ONH (red line; Fig 1A). Five measurements were obtained across the OCT image and averaged for each of the six PBS-injected and twelve cytokine-treated CD1 and NOD eyes. The researcher was masked to the strain and treatment used during OCT thickness measurements to reduce any bias.

### Immunohistochemistry and image analysis

Tissues were collected following CO_2_ asphyxiation of the mice. Immediately after euthanasia, eye globes and attached optic nerves were removed and fixed in 4% paraformaldehyde in 0.1 M PBS, pH 7.4 for 1 h. Eyes and their optic nerves were then washed in PBS and passed through 10, 20 and 30% sucrose solutions before embedding the eyes in optimal cutting temperature medium (Sakura, Netherlands). Eye globes were sectioned at 12 μm thickness. Tissue sections were rinsed in PBS before blocking with 10% normal goat serum and 0.1% TritonX-100 in PBS for 1 h at room temperature. Primary and secondary antibodies were diluted in the blocking solution. Sections were subsequently incubated at 4 °C overnight with anti- ionized calcium-binding adapter molecule 1 (Iba1) (goat polyclonal Iba1; #ab178846; 1:100; Abcam, UK) and anti-glial fibrillary acidic protein (GFAP)-Cy3 used as a marker for astrocytes and hyper-reactive Müller cells (mouse monoclonal GFAP; 1:1000; C9205; Sigma-Aldrich, MO, USA). Sections were then washed in PBS and incubated at room temperature for 2 h in donkey anti-goat Alexa-488 (1:500; Jackson Immuno Research, PA, USA). Nuclei were stained using DAPI (1 μg/mL; D9542; Sigma-Aldrich, MO, USA). Sections were washed and mounted using anti-fade reagent (Citifluor^™^; Electron Microscopy Sciences, PA, USA) and coverslips were sealed with nail polish.

All images were taken with a CCD camera mounted on an Olympus FV1000 confocal laser scanning microscope (Olympus, Japan) and processed using FV-10 ASW 3.0 Viewer and ImageJ software. Three images were taken per eye. Using ImageJ, each image was split into its RGB channels with GFAP in the red, Iba1 in the green, and DAPI in the blue channel. For GFAP immunohistochemical analysis, ImageJ was used to quantify the integrated density (area covered by GFAP labelling × mean grey value) within the optic nerve image. The researcher was masked to the mouse strain and treatment used during GFAP and Iba1 immunofluorescence quantification in order to reduce any bias.

### Statistical analysis

All data are given as arithmetic mean + SEM. Data was first tested for normality using the Shapiro-Wilk test. Since blood glucose data was found to be not normally distributed, it was tested using the nonparametric Mann-Whitney test. All other data was normally distributed, and was tested using Student’s t-test, one-way or two-way ANOVA with *post hoc* Tukey’s test to reduce any bias associated with multiple comparisons. The statistical test used is outlined in each figure legend. p < 0.05 was considered to indicate a statistically significant difference. All statistical analysis was performed in GraphPad Prism 6.

## Results

### Physical comparison of mouse strains

**Fig 2:**
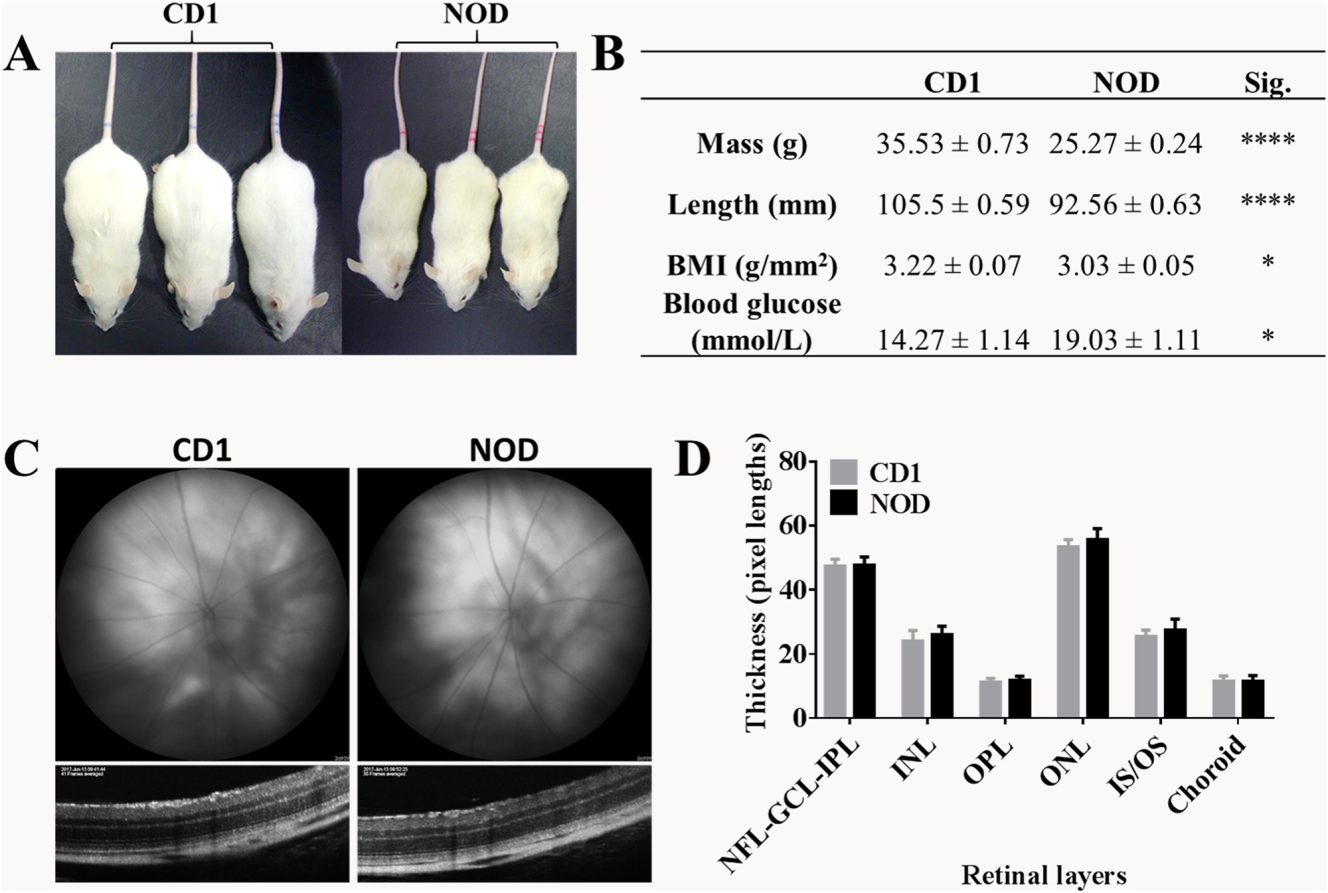
Physical characterisation, retinal integrity and retinal thickness of CD1 and NOD mice. (A) NOD and CD1 mice showing that NOD mice were generally smaller than CD1 mice. (B) A comparison of mass, length, BMI and blood glucose showed that NOD mice were smaller in mass and length (p < 0.0001 for both) and had a significantly lower BMI (p = 0.0279) compared to CD1 mice. However, NOD mice had higher blood glucose levels than CD1 mice (p = 0.0120). Results are expressed as mean ± SEM. Statistical comparisons between NOD and CD1 were carried out using Mann-Whitney test *p ≤ 0.05; ****p < 0.0001; n = 12 eyes per strain. (C) Fundus (top) and OCT (bottom) images of CD1 and NOD mouse retinas showed no differences in retinal vasculature and overall integrity between the two mouse strains. (D) Retinal layer thickness measurements were obtained from OCT images and showed no differences between NOD and CD1 mice at baseline. Results are expressed as mean + SEM; Statistical analysis was carried out using a two-way ANOVA with Tukey’s test for multiple comparisons; n = 12 eyes per strain.

At 15 weeks of age, NOD mice were smaller than CD1 mice (Fig 2A) with NOD mice weighing approximately 10 g less than CD1 mice of the same age (p < 0.0001; Fig 2B) and being significantly shorter (10 mm in length; p < 0.0001). NOD mice had a significantly lower BMI compared to CD1 mice (p = 0.0279). The diabetic state of NOD mice was confirmed using a glucose blood test confirming that they had significantly higher blood glucose levels than non-diabetic CD1 mice (p = 0.0120). It is important to note that the blood glucose levels in NOD mice was likely an underestimate as readings above 27.8 mmol/L were taken as 27.8 mmol/L.

### There were no baseline differences in retinal integrity and thickness between the two mouse strains

Baseline fundus and OCT images from both mouse strains were obtained with representative images shown in Fig 2C and 2D. A comparison of the fundus images (top panel) revealed no obvious differences in retinal macrovascular integrity. Furthermore, OCT images (bottom panel) showed no obvious lesions within the retinal layers. In addition to the qualitative assessment of retinal integrity in fundus and OCT images, the thickness of each retinal layer was measured on OCT images with no significant difference in the thickness of any of the retinal layers at baseline (Fig 2D).

### Intravitreal pro-inflammatory cytokines induced greater evidence of vascular pathology in NOD compared to CD1 mice

**Fig 3:**
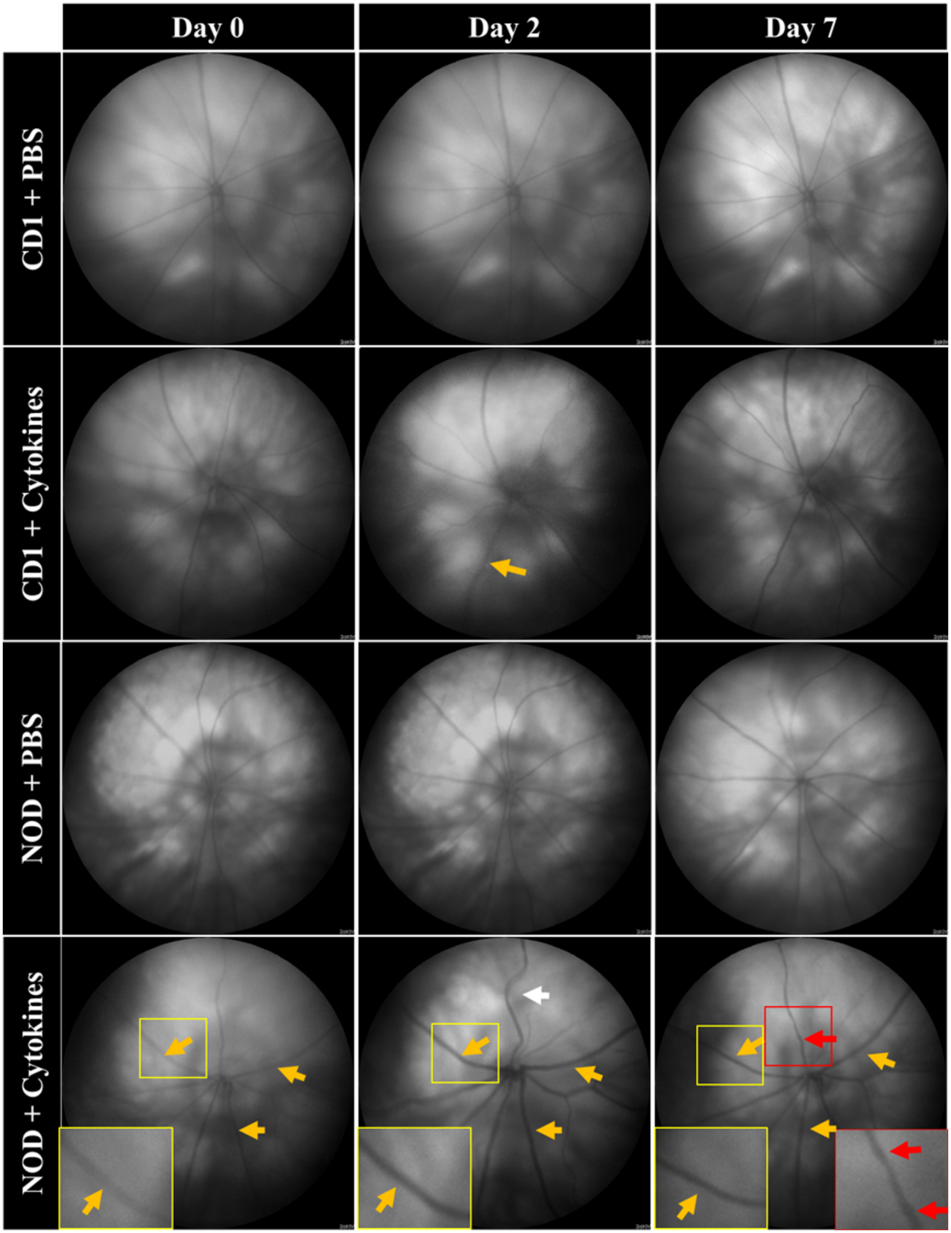
Pro-inflammatory cytokine-induced macrovascular pathology in CD1 and NOD mice. Fundus images showing vascular changes in saline-injected and pro-inflammatory cytokine-treated CD1 and NOD mice. On day 2, vessel dilation (see zoomed images and compare yellow arrows on day 2 with equivalent vessels of the same retina on day 0) increased in both cytokine-treated CD1 and NOD mice while vessel tortuosity (compare white arrow on day 2 with equivalent vessels of the same retina on day 0) was only observed in cytokine-treated NOD mice. Moreover, vessel beading was only seen in cytokine-treated NOD mice and only on day 7 (compare red arrows on day 7 with equivalent vessels of the same retina on day 0).

Pro-inflammatory cytokines, IL-1β and TNF-α, or PBS used as control injection were introduced into the vitreous of both mouse strains and ocular assessments were carried out before injection (day 0) as well as 2 and 7 days after injection. Fundus images revealed no change in vascular morphology in PBS injected CD1 and NOD mice. However, there was an increase in vessel dilation on day 2 compared with baseline in both cytokine treated mouse strains (yellow arrows; Fig 3). Furthermore, vessel tortuosity was found to increase on day 2 but only in cytokine treated NOD mice (white arrow). Fundus images also revealed an increase in vessel beading in cytokine treated NOD mice only on day 7 (red arrows).

### Intravitreal pro-inflammatory cytokine-induced vitreous HRF were greater in number in NOD compared to CD1 mice

**Fig 4:**
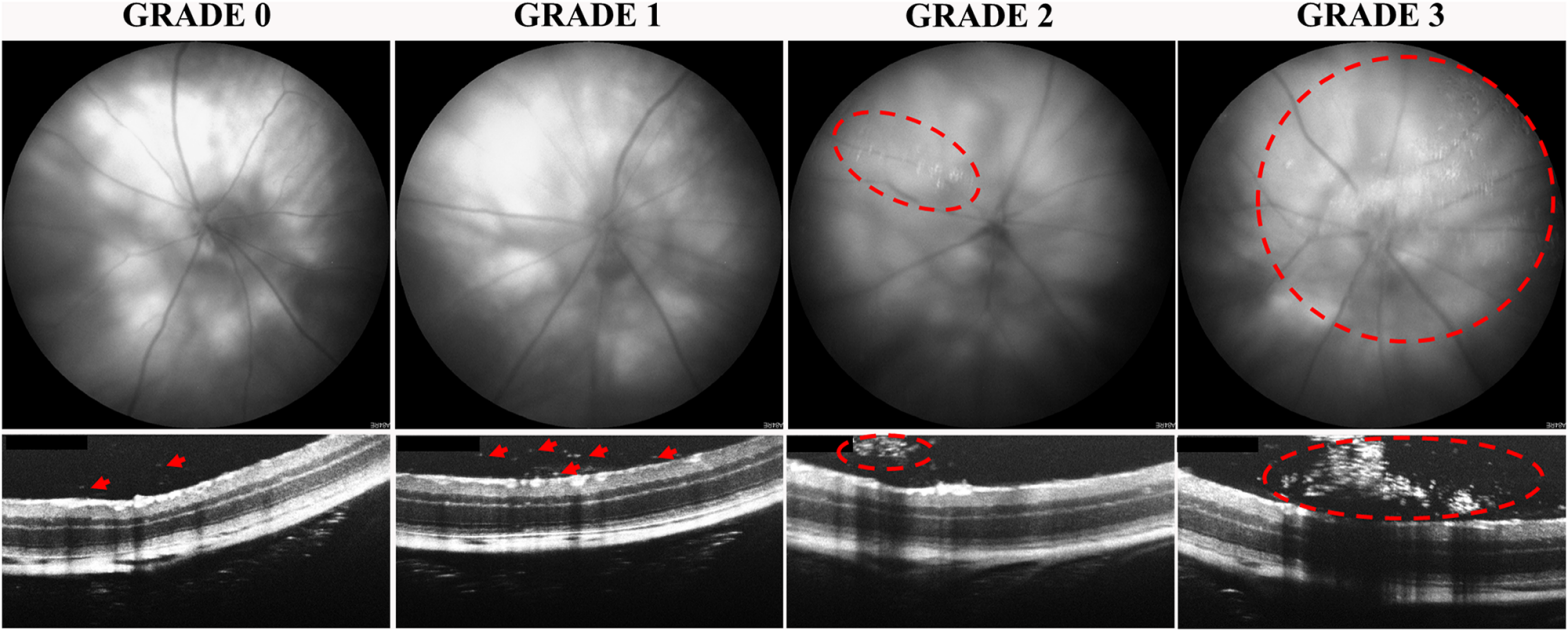
Grading of vitreous HRF based on fundus and OCT images. Grade 0 was characterized by no or very few HRF. Grade 1 showed significantly more HRF in the vitreous (red arrows) seen in OCT images only. Grade 2 and 3 were characterized by clumping of vitreous HRF (red circles) creating a ‘window effect’ that obstructed the view of the underlying retina in both fundus and OCT images. Vitreous HRF were classified as grade 2 if HRF formed clumps obstructing up to 25% of the fundus and OCT image area with grade 3 referring to HRF clumps obstructing more than 25% of the fundus and OCT images.

Vitreous HRF were not observed in PBS injected CD1 or NOD mice (data not included in graph). However, fundus and OCT images revealed the presence of vitreous HRF in both pro-inflammatory cytokine-injected mouse strains. In order to assess the severity of vitreous HRF, a grading scale was developed (Fig 4). Grade 0 represents the presence of no or very few (less than five) HRF in the vitreous OCT image with no impact on the corresponding fundus image. In grade 1, the HRF-containing area in the vitreous is two times bigger than in grade 0 with again no changes seen in the fundus image. Grade 2 represents increased clumping of HRF that create a ‘window effect’ (OCT shadow over the retina) and is visible in the fundus image as a change in contrast which obstructs up to 25% of the fundus and OCT images. Grade 3 is considered to be clumping of HRF with more than 25% of a window effect in both fundus and OCT images.

**Fig 5:**
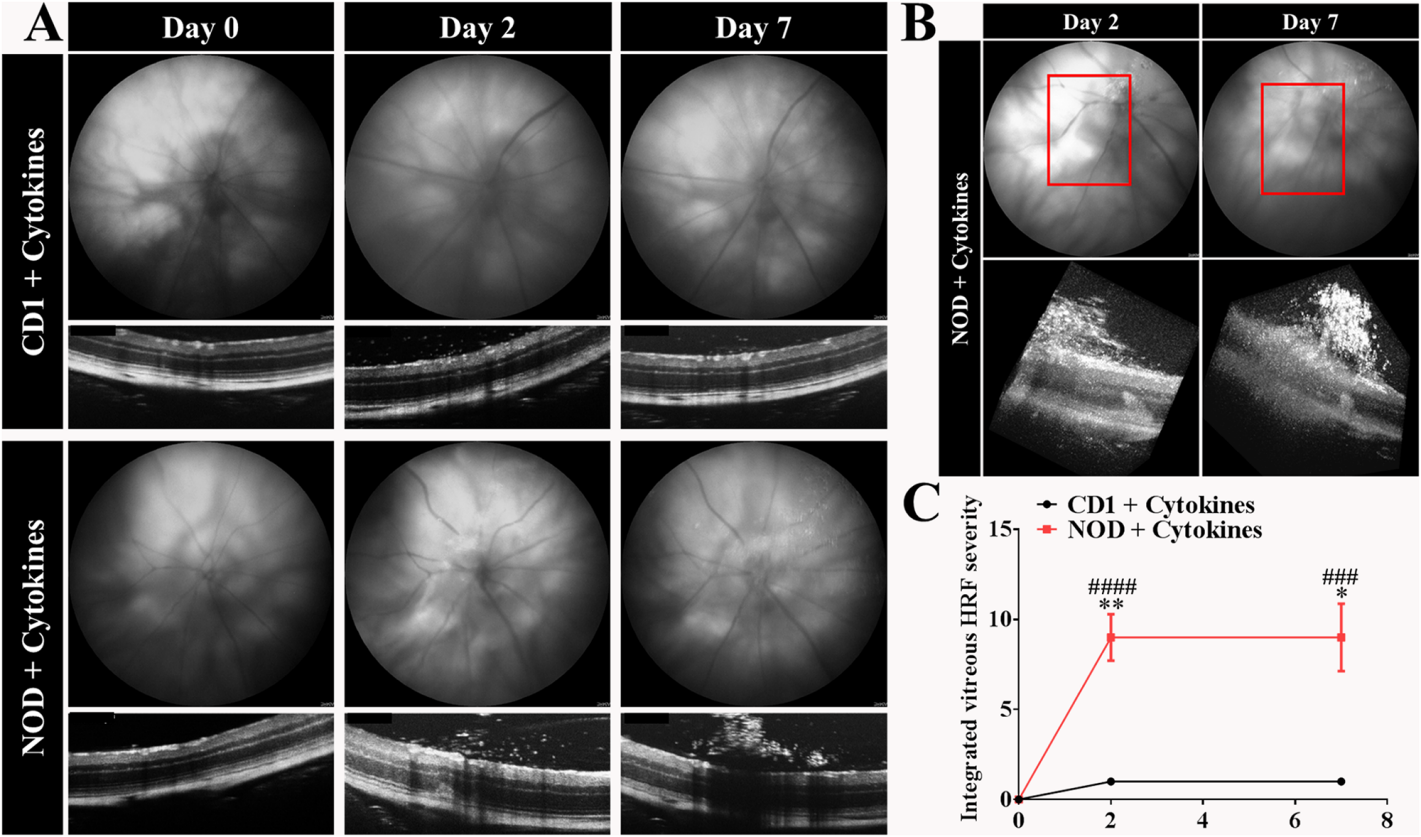
Pro-inflammatory cytokine-induced vitreous hyper-reflective foci in CD1 and NOD mice. (A) Fundus and OCT images showed that vitreous HRF were present in both pro-inflammatory cytokine-treated mouse strains, but were more pronounced in NOD than in CD1 mice. (B) 3D volume OCT images depict the extent of vitreous debris severity in NOD mice on days 2 and 7. (C) The vitreous HRF grading scale was used to compare the vitreous HRF severity between both mouse strains. There was no statistically significant change in severity over time in CD1 mice. However, there was a statistically significant increase in HRF severity in NOD mice at day 2 (p < 0.0001) and day 7 (p = 0.0001) compared to day 0. More importantly, the vitreous HRF severity in cytokine-treated NOD on day 2 (p = 0.0012) and day 7 (p = 0.0428) was significantly higher than in cytokine-treated CD1 eyes. Results are expressed as mean ± SEM; Statistical comparisons were carried out using two-way ANOVA with Tukey’s multiple comparisons test. #denotes statistically significant differences compared to day 0. ^###^p ≤ 0.001; ^####^p < 0.0001. *denotes significant differences between cytokine-injected CD1 and NOD mice. *p ≤ 0.05; **p ≤ 0.01; n = 12 eyes per strain.

There was an increase in vitreous HRF severity in cytokine-treated NOD mice at day 2 (p < 0.0001) and day 7 (p = 0.0001) compared to day 0 (Fig 5). However, there was no statistically significant difference in vitreous HRF severity over time in cytokine-treated CD1 mice. More importantly, the severity of vitreous HRF was higher in NOD compared to CD1 mice at day 2 (p = 0.0012) and day 7 (p = 0.0428) post-cytokine injection.

### Intravitreal pro-inflammatory cytokine-induced intra-retinal abnormalities and thickening of the NFL, GCL and IPL was greater in NOD compared to CD1 mice

**Fig 6:**
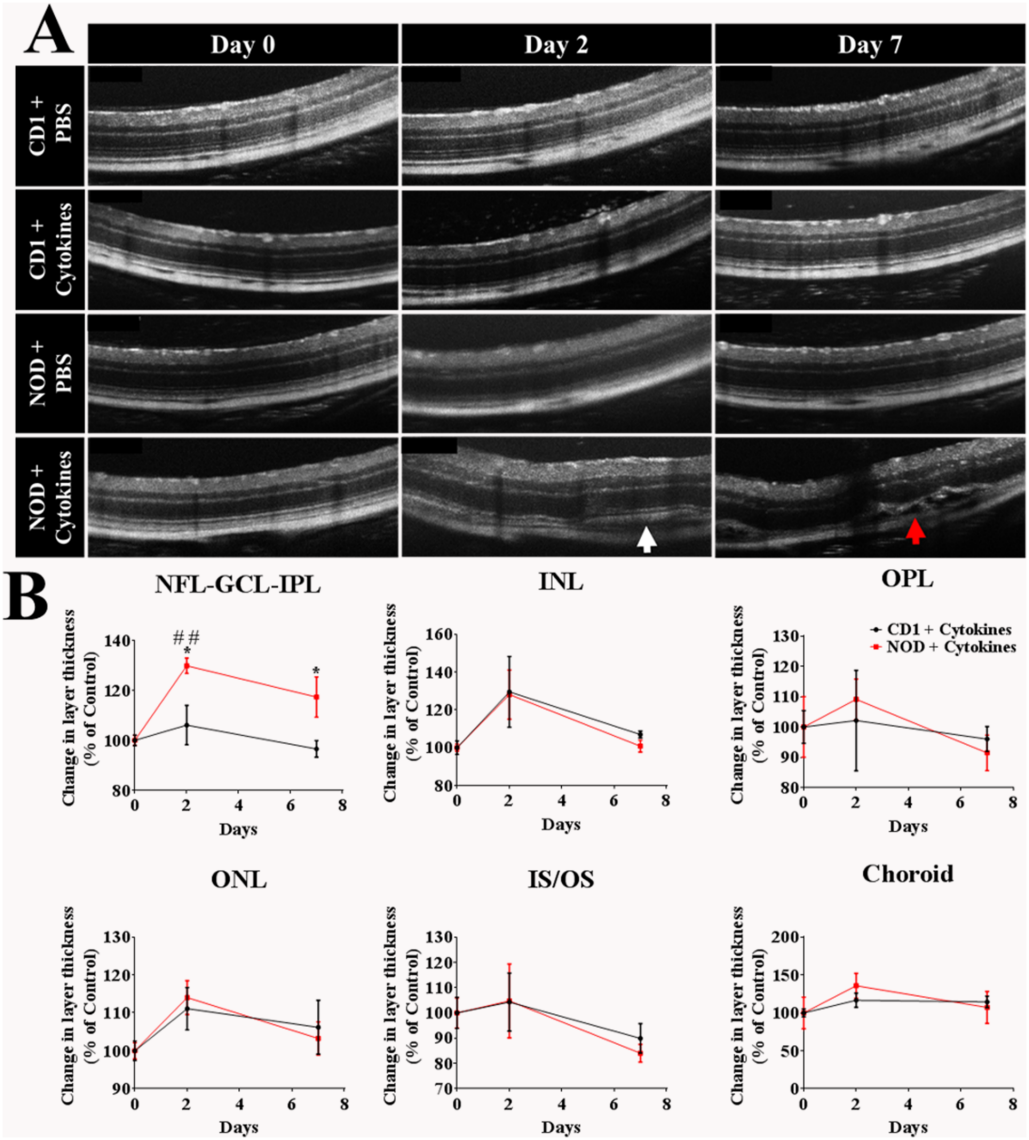
Pro-inflammatory cytokine-induced intra-retinal hyper-reflective foci in CD1 and NOD mice. (A) OCT images revealed that saline injection did not affect retinal layer integrity in either mouse strain. Pro-inflammatory cytokine injection did not affect retinal layer integrity in CD1 mice. However, cytokine-injection in NOD mice resulted in severe disruption of the IS/OS on day 2 (white arrow) and retinal oedema on day 7 (red arrow). (B) Retinal layer thickness was quantified from OCT images and expressed as a percentage of the baseline thickness. The thickness of the NFL-GCL-IPL increased over time in cytokine-treated NOD mice and this increase was significant on day 2 compared to NOD baseline values (p = 0.0025) and CD1 mice at day 2 (p = 0.0188). At day 7, cytokine-treated NOD mice again showed thicker NFL-GCL-IPL compared to cytokine-treated CD1 mice (p = 0.0410). At both day 2 and day 7, no significant increase was found in the thickness of the INL, OPL, ONL, IS/OS, or choroid in NOD or CD1 compared to their baseline Results are expressed as mean ± SEM; Statistical comparisons were carried out using two-way ANOVA with Tukey’s multiple comparisons test. #denotes statistically significant differences compared to day 0. ^##^p ≤ 0. *denotes significant differences between cytokine-injected CD1 and NOD mice. *p ≤ 0.05. n = 12 eyes per strain.

OCT images showed the presence of intra-retinal abnormalities in cytokine injected NOD mice only with an increase in retinal layer disruption, particularly in the inner segment/outer segment of photoreceptors (IS/OS) on day 2 (white arrow; Fig 6A). Furthermore, there was an increase in retinal oedema by day 7 (red arrow, Fig 6A). PBS injected CD1 and NOD mice as well as cytokine injected CD1 mice did not show any intra-retinal abnormalities.

Changes in retinal thickness were then quantitatively assessed from OCT images (Fig 6B). Retinal layer thickness did not change in PBS injected CD1 and NOD mice over time (data not shown in graph). However, a change in layer thickness was seen in NOD mice but only in the NFL-GCL-IPL following intravitreal cytokine administration. On day 2, there was a significant increase in NFL-GCL-IPL thickness in cytokine-treated NOD mice compared to baseline (p = 0.0025) and cytokine-treated CD1 mice on day 2 (p = 0.0188). At day 7, cytokines had also induced thickening of the NFL-GCL-IPL in NOD compared to CD1 mice with a mildly significant difference between day 7 and day 0 time points in both mouse strains (p = 0.0410). The TUNEL apoptosis assay revealed that the increase in layer thickness did not correlate with a reduction in thickness of other retinal areas as no cell death was observed in either cytokine-treated CD1 or NOD mice (data not shown). Furthermore, histological analysis (Haematoxylin & Eosin) revealed that the majority of thickness increase was due to swelling in the IPL (S1 Fig). At both day 2 and day 7, there was no significant increase in the thickness of the INL, OPL, ONL, IS/OS, or choroid in NOD or CD1 compared to their baseline value.

### Intravitreal pro-inflammatory cytokines induced retinal HRF in NOD mice

**Fig 7:**
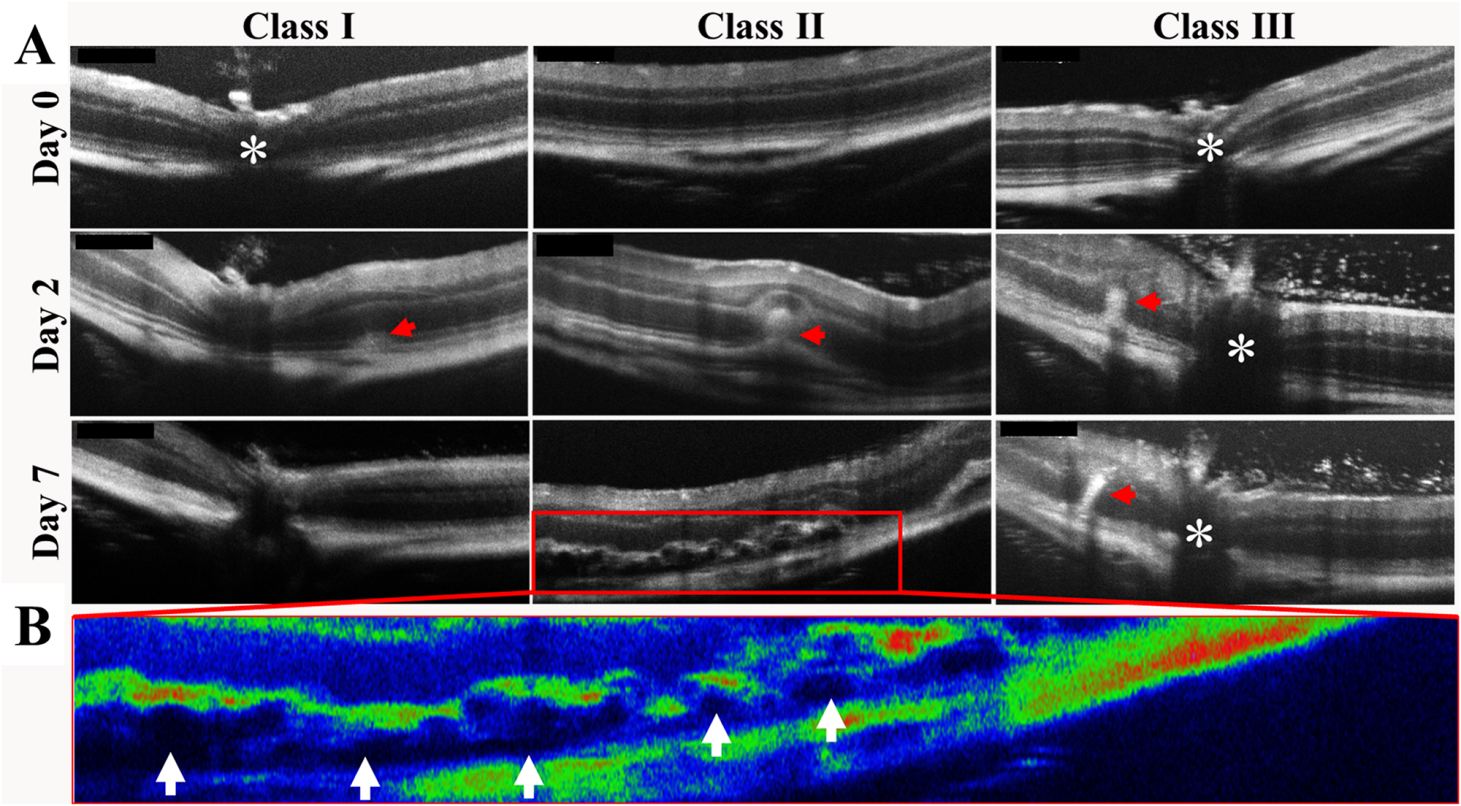
Pro-inflammatory cytokine-induced changes in retinal layer thickness in CD1 and NOD mice. (A) Different classes of intra-retinal HRF (red arrows) were observed within the ONL in pro-inflammatory cytokine-injected NOD mouse retinas. The ONH is indicated by asterisks. (B) A pseudo-colour OCT image of class II graded retinal HRF seen in (A) showing the hypo-reflective regions of sub-retinal fluid deposition between the IS/OS layers and the RPE (white arrows).

OCT images revealed that retinal HRF were present in cytokine treated NOD mice only (Fig 7A). Three different classes of retinal HRF, observed within the ONL (red arrows, Fig 7A), are defined in Table 1. In class I, retinal HRF were small, located in the IS/OS layer and did not distort any of the overlaying layers. By day 7, class I retinal HRF resolved leaving behind no obvious pathologies. In class II, retinal HRF located in the ONL were much larger and distorted the overlaying OPL with no obvious changes to other layers of the inner retina. By day 7, class II HRF were no longer visible but were replaced by sub-retinal fluid that accumulated between the IS/OS layer and the RPE (white arrows; Fig 7B). Class III HRF were columnar in shape, occupied the ONL, and extended to the OPL. By day 7, class III HRF had grown to breach the OPL and extend into the INL and IPL, while also being associated with numerous smaller HRF in the inner retina and vitreous.

**Table 1:** Description of the different classes of HRF in cytokine-treated NOD mice.

Classes	Description	
	Day 2	Day 7
**Class I**	HRF seen in the IS/OS	HRF disappeared
**Class II**	HRF seen within the IS/OS and ONL and distorted OPL	HRF disappeared, appearance of sub-retinal fluid
**Class III**	Columnar HRF seen within the ONL and distorted OPL	Columnar HRF remained and extended into the inner retinal layers

### Intravitreal pro-inflammatory cytokine-induced retinal GFAP over-expression was greater in NOD compared to CD1 mouse retinas

**Fig 8:**
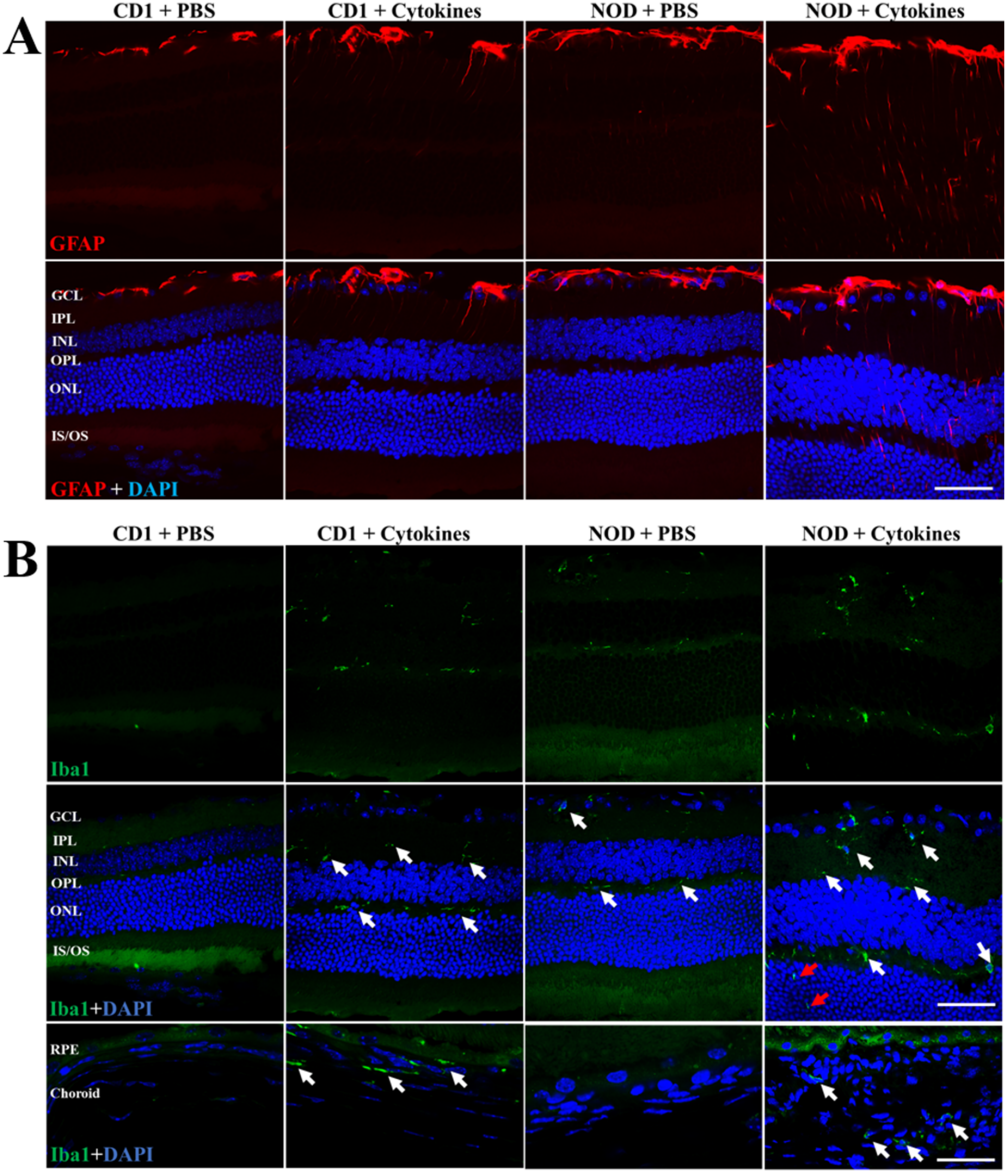
Pro-inflammatory cytokine-induced changes in retinal expression of GFAP and Iba-1 in CD1 and NOD mice. (A) Immunohistochemical images showing GFAP expression in PBS-injected or cytokine-treated CD1 and NOD mice. GFAP labelling was evident only within the GCL in PBS-injected CD1 and NOD mouse retinas. In cytokine-treated CD1 retinas, GFAP expression was seen in the GCL but also extended to the IPL. Cytokine-treated NOD retinas, on the other hand, showed GFAP expression extending from the GCL to the ONL. As with OCT analysis, retinal layers of cytokine-treated NOD mice appeared much thicker than those of other treatment groups. (B) Immunohistochemical images showing the presence of Iba1-positive cells within the GCL, IPL, and INL of cytokine-treated CD1 as well as PBS- and cytokine-injected NOD mice (white arrows). Moreover, cytokine-treated NOD retinas revealed Iba1-positive cells (red arrows) within the OPL which were not observed in other groups. Iba1-positive cells were absent in the choroid of PBS-injected CD1 and NOD mice. However, Iba1-positive cells were observed in the choroid of cytokine-treated CD1 and NOD mice as indicated by white arrows. GCL = Ganglion cell layer; IPL = Inner plexiform layer; INL = Inner nuclear layer; OPL = Outer plexiform layer; ONL = Outer nuclear layer; IS/OS = Inner segment/outer segment; RPE = retinal pigment epithelium. Scale bar: 25 μm.

GFAP was used as a marker of glial reactivity following intravitreal administration of pro-inflammatory cytokines (Fig 8A). At 7 days post-injection, cytokine-treated NOD mice displayed increased GFAP labelling spanning from the GCL to the ONL while cytokine-treated CD1 mice showed increased GFAP expression spanning up to the IPL only. In contrast, GFAP expression was normal and restricted to the GCL in both PBS-injected CD1 and NOD mice.

### Intravitreal pro-inflammatory cytokines induced infiltration of Iba1-positive cells into the ONL in NOD but not CD1 mice

Iba1-positive cells were present in the GCL, IPL and OPL of NOD mice injected with cytokines, NOD mice injected with PBS, and CD1 mice injected with cytokines (Fig 8B). Additionally, cytokine injected NOD mice displayed infiltration of Iba1-positive cells into the ONL. Furthermore, results showed that Iba1-positive cells were absent in the choroid of NOD + PBS and CD1 + PBS mice, but were observed in the choroid of cytokine-treated NOD and CD1 mice.

### Intravitreal pro-inflammatory cytokines increased GFAP expression and process-containing Iba1-positive cells in the optic nerve of NOD but not CD1 mice

**Fig 9:**
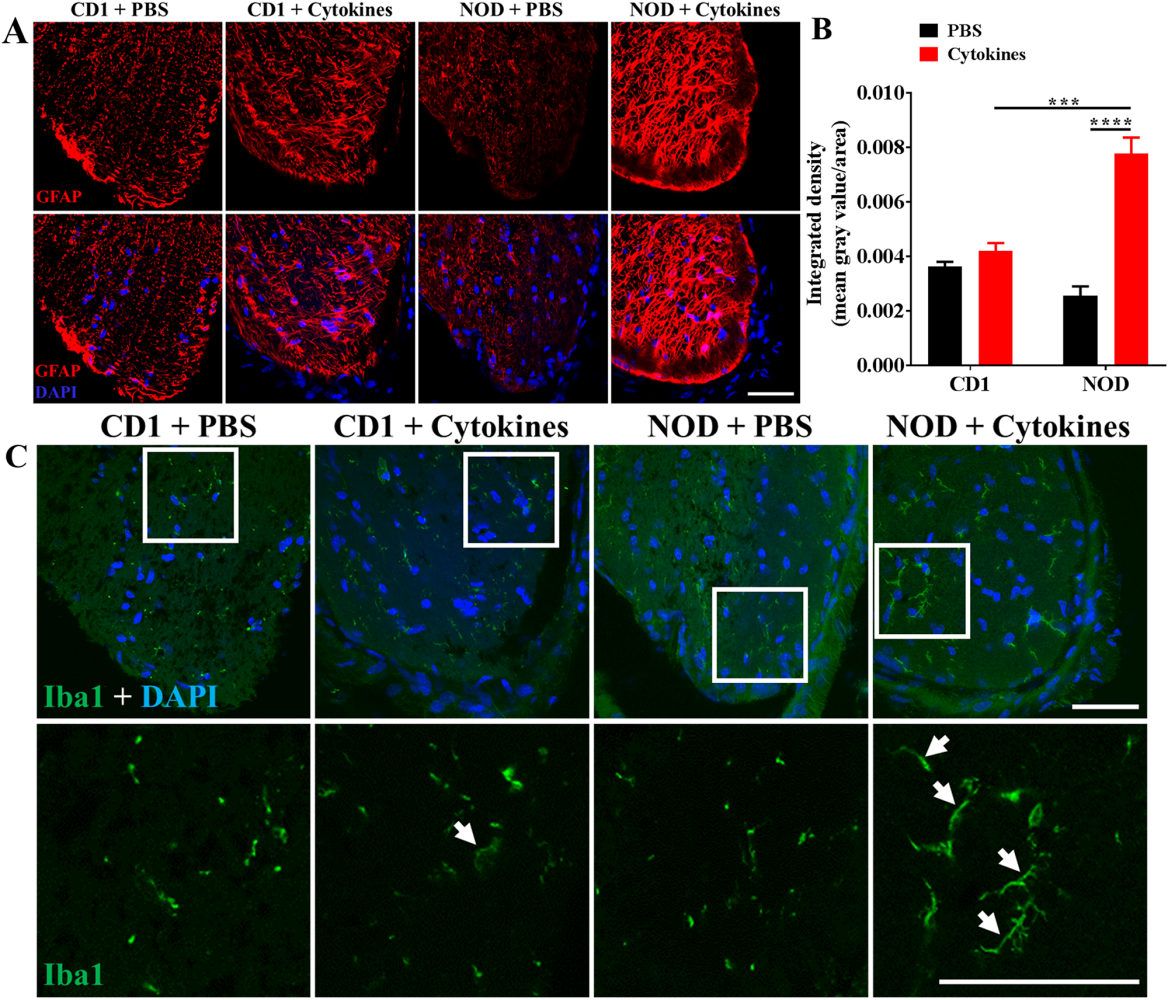
Pro-inflammatory cytokine-induced changes in retinal expression of GFAP and iba-1 in CD1 and NOD mice. (A) Immunohistochemical images showing GFAP expression in the optic nerve of PBS-injected and cytokine-treated CD1 and NOD mice. GFAP labelling was evident in all treatment groups but appeared highest in cytokine-treated NOD mice. (B) Quantification of the integrated density of GFAP in the optic nerve of PBS-injected and cytokine-treated CD1 and NOD mice revealed that GFAP expression was higher in cytokine-treated compared to PBS-injected NOD (p < 0.0001) and cytokine-treated CD1 mice (p = 0.0003). (C) Immunohistochemical images showing the presence of Iba1-positive cells within the optic nerve in all treatment groups. Compared to PBS-injected groups, Iba1-positive cells in cytokine-treated eyes displayed long, extended cellular processes (white arrows). White boxes in the top row have been zoomed in on the bottom to highlight the differences in Iba1-positive cells. Statistical comparisons were carried out using two-way ANOVA with Tukey’s multiple comparisons test. Scale bar: 25 μm; Data presented as mean + SEM. ***p ≤ 0.001; ****p ≤ 0.0001; n = 6 eyes for PBS injected groups; n = 12 eyes for cytokine-treated groups.

Cytokine-treated NOD mice showed significantly higher GFAP expression in the optic nerve relative to PBS treated NOD mice (p < 0.0001) or even cytokine treated CD1 mice (p = 0.0003) (Fig 9B). Iba1-positive cells were also seen in the optic nerve, whit their shape different from cytokine-treated groups (Fig 9C). The optic nerve of cytokine-treated CD1 and NOD mice contained Iba1-positive cells with long extended processes (white arrows; Fig 9C) compared to PBS-injected controls where cells appeared small and condensed. PBS and cytokine-injected CD1 mice showed no difference in GFAP expression in the optic nerve (Fig 9A).

## Discussion

Clinically, DR is associated with endothelial cell death, pericyte loss, BRB breakdown, intra-retinal microvascular abnormalities, retinal and optic nerve neovascularisation and macular oedema [2,20–24]. Most animal models of DR do not represent these clinical signs. However, in this study, we provide evidence that a single intravitreal injection of pro-inflammatory cytokines, IL-1β and TNF-α, may be sufficient to induce clinical signs of DR in diabetic NOD mice. Prior to the intravitreal cytokine injection, we conducted baseline assessments of both non-diabetic CD1 and diabetic NOD mice with CD1 mice being significantly larger and heavier than the NOD mice. This weight difference is not surprising as it has previously been reported that diabetic mice, particularly with type 1 diabetes, tend to weigh less than their non-diabetic controls due to their metabolic imbalance [25]. It is noteworthy that CD1 mice, the genetic background strain for NOD mice, showed higher blood glucose levels than expected. This could be because non-fasting blood glucose levels were measured in this study or, as suggested in other studies, glucose levels below 15 mmol/l are mild forms of the diabetic condition [26]. Nonetheless, NOD mice still showed significantly higher blood glucose levels compared to CD1 mice. Despite being hyperglycaemic, NOD mice showed no significant difference in retinal thickness and vascular integrity compared to CD1 mice at baseline. This is in line with studies suggesting that NOD mice only show subtle changes in retinal vascularisation but do not display signs of proliferative DR normally visible during fundus and OCT examination in humans [15,16].

We tested the hypothesis that introducing pro-inflammatory cytokines into the eye of NOD mice may be able to induce ocular lesions resembling signs of DR. Interestingly, despite receiving the same dose of cytokines, CD1 mice did not show as many of the signs seen in NOD mice. The ocular signs observed in CD1 mice included vessel dilation, vitreous HRF (average integrated vitreous HRF severity of 1 on day 7), retinal and optic nerve astrogliosis and microgliosis (Table 2). While these are signs seen in DR, they are not unique to the condition and can also be observed in other intraocular inflammatory diseases such as uveitis [27] and glaucoma [28]. For instance, glaucoma is associated with pronounced ONH inflammation, astrogliosis and microglial activation [28]. As expected, introduction of cytokines into the vitreous induced vitreous inflammation (vitritis) and evoked an inflammatory response in surrounding ocular tissues. This is in line with a study by Ferrick and colleagues where ocular inflammation was stimulated by intravitreal IL-8 and IL-1 injection in rats [29].

**Table 2:** Summary of findings from the present study and supporting clinical evidence from clinical DR studies.

Pathologies	Summary of results from present study		Supporting evidence from clinical studies
	CD1	NOD	
**Vessel dilation**	**+**	**++**	[30]
**Vessel beading**	**-**	**+**	[30]
**Vitreous HRF**	**+**	**++**	[35]
**Retinal HRF**	**-**	**+**	[36,37]
**Sub-retinal fluid**	**-**	**+**	[38]
**Thickening of inner retinal layers**	**-**	**+**	[39,40]
**Gliosis and microglia activation**	**+**	**+**	[41–43]
**Optic nerve astrocytosis and microglia activation**	**+**	**++**	[44]

- indicates absence of pathology; + indicates presence of pathology; ++ indicates higher levels of pathology

The pro-inflammatory cytokines had a more severe effect in diabetic NOD mice. The additional signs observed in NOD mice included vessel beading, more severe vitreous HRF (average integrated vitreous HRF severity of 9 on day 7), retinal HRF, sub-retinal fluid accumulation, increased NFL-GCL-IPL thickness, and more severe retinal and optic nerve astrogliosis and microgliosis (Table 2). These are all clinical signs reported in patients with severe or proliferative DR as referenced in Table 2. Pronounced vessel dilation and beading, both of which were prominent in cytokine-treated NOD mice, are characteristic signs of pericyte loss, endothelial cell death, and BRB breakdown [30]. Vitreous HRF, also known as vitreous inflammatory cells, have been reported in patients with severe proliferative DR [31,32], and in animal models of ischemic optic neuropathy, these HRF are thought to be macrophages [33]. Authors of these studies, and others, believe that the presence of HRF within the vitreous reflects the infiltration of immune cells that contribute to vascular breakdown and endothelial cell damage [32,34]. The fact that macrophage infiltration is increased following cytokine injection in the present study also supports the notion of immune cell involvement in the pathogenesis of the disease. While vitreous HRF were seen in both NOD and CD1 mice, we found that the total proportion of eyes with vitreous HRF as well as the severity was much higher in diabetic NOD compared to the non-diabetic CD1 mice. This suggests that the signs observed are not simply a result of inflammation but reflect an interplay between inflammation and hyperglycaemia, which may be important in the development of microvascular complications that arise in diabetic patients.

Unlike vitreous HRF, retinal HRF were only observed in cytokine injected NOD mice. These were divided into three classes based on their morphology and the resulting pathology on day 7. Interestingly, the most common class of retinal HRF, Class II, resulted in deposition of fluid between the IS/OS and the RPE by day 7. This is significant as retinal oedema is a classic sign of more advanced DR [45]. In humans, the oedema usually occurs at the macula, the area critical to high quality visual function. However, rodents lack a macula and the oedema was observed throughout the entire retina instead, from the periphery to areas around the ONH. In addition to sub-retinal fluid accumulation, there was a thickening of the IPL that suggests oedema within Müller cells [39]. More studies are required to confirm this and its significance for disease progression.

Increased GFAP expression has long been used as a marker of retinal astrogliosis in models of DR [46–49]. Unsurprisingly, we found increased GFAP expression in both NOD and CD1 mice following cytokine injection, suggesting that the cytokine insult induced retinal stress in both mouse strains. However, the cytokine effect on GFAP expression was more pronounced in NOD than in CD1 mice. The increase in GFAP was not restricted to the inner retina alone but was also seen in the ONH with statistically higher GFAP labelling seen in cytokine-treated NOD mice compared to all other groups.

This investigation has shown that DR models incorporating hyperglycaemia and inflammation seem to result in a more reliable model of DR, where retinal damage is pronounced or accelerated in the presence of inflammation [50,51]. This study, therefore, supports the growing literature evidence that inflammation is important for the disease development and is not just a result of hyperglycaemia. This is further supported by studies showing that intraocular inflammatory diseases are linked to DR progression in diabetic patients [7]. Therefore, while it is generally believed that prolonged diabetes is associated with increased inflammation leading to DR, it is also possible that new or existing inflammatory conditions of the eye can accelerate the development of DR. This could be particularly important in understanding the reason why there are large variations in the time between the onset of diabetes and the development of DR in the overall population.

A possible limitation to the use of NOD mice to explain mechanisms of DR could be their proneness to autoimmunity due to a genetic defect that causes cytokine dysregulation and a reduced IL-1 response [52]. Similarly, type I diabetes is associated with several autoimmune diseases in humans [53]. Studies by Serreze and colleagues reported that macrophages derived from NOD mice are poorly developed resulting in defective cytokine regulation [54,55]. Furthermore, Fan and colleagues found that macrophages from NOD mice have suppressed responses to TNF-α and liposaccharide stimulation, particularly in terms of IL-1β expression, in line with Serreze and colleagues. In contrast, some studies suggest that the pathology of NOD mice is associated with increased levels of TNF-α and IFN-γ in the spleen, though this is yet to be studied in the eye [56]. Nonetheless, the fact that cytokine injected NOD mice appear to display key features suggestive of DR means that this model might be used to study aspects of the DR pathology, albeit keeping its limitations in mind.

In conclusion, intravitreal inflammatory cytokine injection in NOD mice appears to provide a novel model for the study of signs consistent with the development of DR in humans. The model may provide an opportunity to further study the molecular basis of the disease in order to understand the disease pathology. While the present study focused on the eye, it is plausible that inflammation plays a similar role in exacerbating hyperglycaemia-mediated injury in other microvascular complications of diabetes such as nephropathy and neuropathy, thus the concept could be expanded for these indications.

## Supporting information

S1 Fig Histological images of CD1 and NOD mouse retinas following cytokine injection. Retinal sections stained with H&E showed an increase in IPL thickness in both CD1 and NOD mice following intravitreal pro-inflammatory cytokine administration. However, the increase in IPL thickness was more pronounced in NOD + cytokines compared to CD1 + cytokines.
(TIF)
