# Supplementary figures and images for: Intravitreal pro-inflammatory cytokines in non-obese diabetic mice: Modelling signs of diabetic retinopathy
Source: PLoS One. 2018 Aug 22;13(8):e0202156. doi: 10.1371/journal.pone.0202156 (PMC6105000; doi:10.1371/journal.pone.0202156)

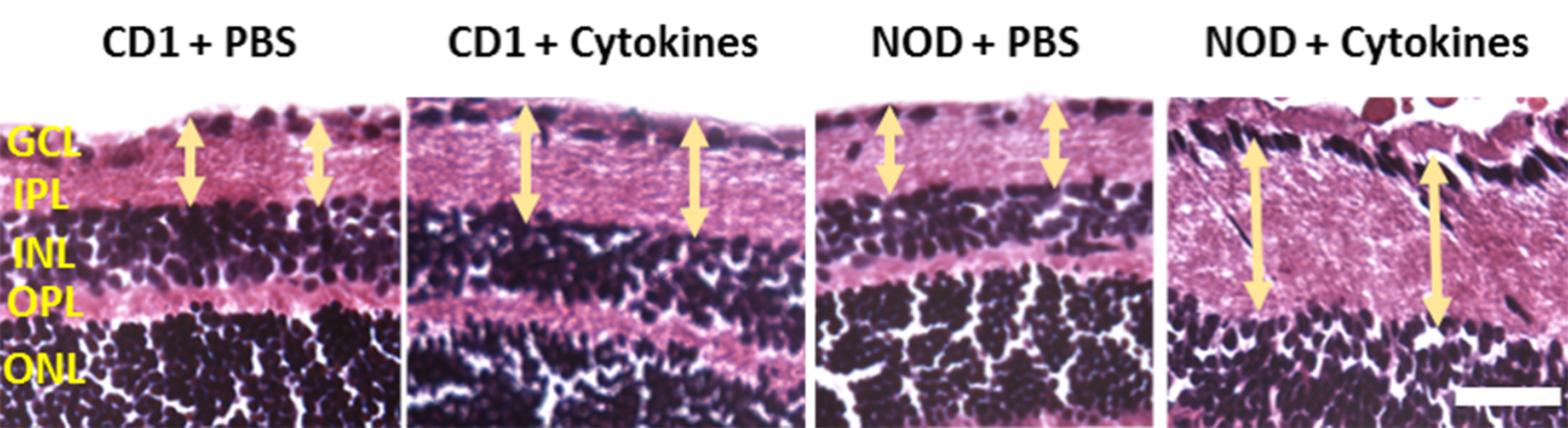

Supplement: S1 Fig — Retinal sections stained with H&E showed an increase in IPL thickness in both CD1 and NOD mice following intravitreal pro-inflammatory cytokine administration. However, the increase in IPL thickness was more pronounced in NOD + cytokines compared to CD1 + cytokines. (TIF) [file pone.0202156.s001.tif]
